# Quercetin liposomes ameliorate streptozotocin-induced diabetic nephropathy in diabetic rats

**DOI:** 10.1038/s41598-020-59411-7

**Published:** 2020-02-12

**Authors:** Lixia Tang, Ke Li, Yan Zhang, Huifang Li, Ankang Li, Yuancheng Xu, Bing Wei

**Affiliations:** 1 Department of Endocrine, The First People’s Hospital of Yongkang, Jinhua, 321300 P.R. China; 2 Department of Pathology, Zhucheng Maternal and Child Health Hospital, Weifang, 262200 P.R. China; 3 Department of Pharmacy, The First People’s Hospital of Yongkang, Jinhua, 321300 P.R. China; 4 Department of Pathology, The First People’s Hospital of Yongkang, Jinhua, 321300 P.R. China; 5 Department of Orthopedics, The First People’s Hospital of Yongkang, Jinhua, 321300 P.R. China

**Keywords:** Diabetes, Drug development, Chronic kidney disease

## Abstract

The effects of quercetin liposomes (Q-PEGL) on streptozotocin (STZ)-induced diabetic nephropathy (DN) was investigated in rats. Male Sprague Dawley rats were used to establish a STZ induced DN model. DN rats randomly received one of the following treatments for 8 weeks: blank treatment (DN), free quercetin (Que), pegylated liposomes (PEGL) and pegylated quercetin liposomes (Q-PEGL). A group of healthy rats served as the normal control. The fasting blood glucose (FBG), body weights (BWs), renal hypertrophy index (rHI), serum and urine biochemistry, renal histopathology, oxidative stress and immunohistochemical measurements of AGEs were analyzed to compare the effect of different treatments. Que and Q-PEGL significantly improved DN biochemistry and pathological changes, although the treated rats still had some symptoms of DN. The therapeutic effect of Q-PEGL surpassed that of Que. Pegylated quercetin liposomes allow maintaining higher quercetin concentrations in plasma than non-encapsulated quercetin. In conclusion the use of quercetin liposomes allows to reduce disease symptoms in a rat model of DN.

## Introduction

Diabetes mellitus (DM) is a metabolic disorder characterized by hyperglycemia due to impaired body’s ability to produce or respond to the hormone insulin^1^. Diabetic nephropathy (DN) is a microvascular complication of DM and causes long-term or end-stage renal disease^2^. Multifactorial interaction between lipid disorders, oxidative stress, renal hemodynamic changes, polyol activation, inflammatory pathways, and mitogen-activated protein kinase signaling pathways are involved in the pathophysiology of DN^3,4,7^. Therefore, strong antioxidants could potentially serve as treatments to diabetes related diseases^5^ Quercetin (C_15_H_10_O_7_,3′,4′,5,7-pentahydroxyflavone) is a potent dietary bioflavonoid found in diverse fruits, seeds, and vegetables such as legumes, apples, and chili peppers^4–7^. Pharmacological studies in humans show that quercetin has multiple biological functions including circulation system protection, anti-allergic, anti-inflammatory, anti-cancer, anti-diabetes, and cataract prevention^7^. Another important attribution of quercetin is to reduce aldose reductase, which is an enzyme that converts glucose to sorbitol through polyol pathway^5^.

Natural quercetin has poor water solubility, therefore, quercetin liposomes are laboratory-prepared to improve its solubility and *in vivo* absorbability^7–9^. Liposomes are the most studied particle carrier systems allowing sustained release, and have the potential of enhancing the oral bioavailability of proteins and peptides^10^. Liposomes are small spherical lipid vesicles, composed of phospholipids and cholesterol, characterized on size, number of lamellae, and inner/outer phases^11,12^. Liposomes have good cell compatibility, reduce drug toxicity, improve drug stability and function for a long time^13^, and have been used to deliver drugs including antibiotic, antifungal, and cytotoxic agents^14^. Coating inert biocompatible polymers, such as PEG, on the liposome surface will form a protective layer over the liposome surface, which slows down liposomes recognition and clearance, and extends liposome circulation time and provides slow release of an encapsulated drug^9,15^. Yuan *et al*.^9^ utilized liposomes for delivery of quercetin to study anti-tumoral properties. Yue *et al*.^13^ also developed coenzyme Q10-loaded liposomes to treat DN.

Streptozotocin (STZ) is used to induce diabetic kidney injury with hyperuricemia, hyperlipidemia and inflammation^2,16^. In the present study, we evaluated the effects of quercetin liposomes on STZ-induced DN rats through comparing the histopathological and biochemical alterations among different treatment groups.

## Results

### Pharmacokinetics and kidney distribution of free Que or Q-PEGL

**Figure 1:**
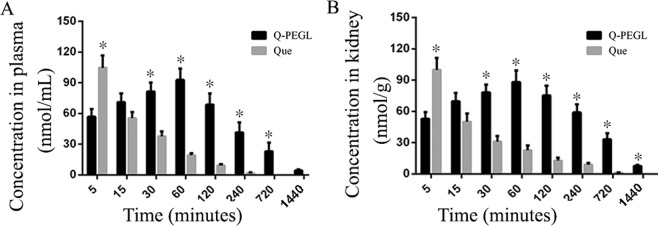
Distribution of quercetin liposomes and free quercetin in DN rats. Rats were intragastrically treated with free Que (50 mg/kg) or Q-PEGL (200 mg/kg) dissolved in 0.9% sodium chloride. The quercetin in plasma and kidney were detected by high-performance liquid chromatography. (**A**) concentration-time curve of quercetin in plasma both Q-PEGL and free Que; (**B**) kidney distribution of free Que and Q-PEGL at different time points (5, 15, 30, 60, 120, 240, 720, and 1,440 min). *Indicates a difference of P < 0.05.

The maximum concentration of quercetin in the plasma was 92.77 ± 11.21 nmol/ml for the Q-PEGL group at 60 minutes and was 104.53 ± 12.08 nmol/ml for the Que group at 5 minutes (Fig. 1A). The maximum concentration of quercetin in the kidney was 88.03 ± 11.20 nmol/g for the Q-PEGL group at 60 minutes and was 99.80 ± 11.37 nmol/ml for the Que group at 5 minutes (Fig. 1B). In the Q-PEGL group, quercetin was still detectable at 1,440 minutes in the plasma and kidney. The concentrations of quercetin were relatively high in plasma and kidney in rats treated by free quercetin (Que group) at 5 minutes, and the concentration of quercetin declined with time. After 15 minutes the quercetin concentration was higher in the Q-PEGL than in the Que group (Fig. 1).

### Changes in BWs, FBG, rHI, and 24-h urinary protein of rats with STZ induced DN

**Figure 2:**
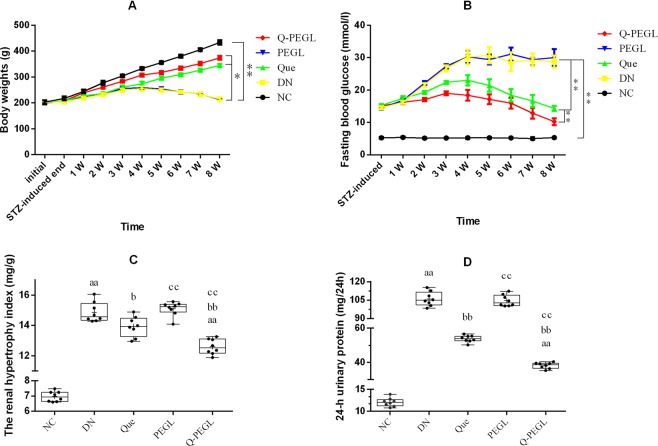
The mean BWs (**A**), FBG (**B**), rHI (**C**) and 24-h urinary protein (**D**) of all groups (*n* = 8). Data were presented as the mean ± S.D. ^a^*P* < 0.05, ^aa^*P* < 0.01 *versus* control group. ^b^*P* < 0.05, ^bb^*P* < 0.01*versus* DN group. ^c^*P* < 0.05, ^cc^*P* < 0.01 *versus* Que group. NC: normal control; DN: blank treatment; Que: free quercetin 50 mg/kg/day; PEGL: liposomes dissolved in 0.9% sodium chloride 150 mg/kg/day; Q-PEGL: Pegylated quercetin liposomes 200 mg/kg/day. *Indicates a difference of P < 0.05, and **indicates a difference of P < 0.01.

The characteristics of STZ induced DN rat are summarized in Fig. 2. The body weight was decreased in the DN rats but increased in the normal control group (*P* < 0.01). The body weight of all groups increased after STZ induction until week 4, from where the body weight started to decrease in the DN group and the PEGL group, while kept increasing in the rest three groups. At the end of the treatment period (8 weeks after administration of STZ), body weights in NC group and PEGL group were significantly lower than those of the other groups (*P* < 0.01). No significant difference was detected between the DN and the PEGL groups (*P* > 0.05). The body weight of Q-PEGL rats was significantly higher than that of the other groups (P < 0.01), except that it was lower than that of the NC group (P < 0.01).

At the end of the STZ injection, FBG level was significantly increased in DN model rats compared to NC rats (P < 0.01). Treatment reduced FBG level in the Que and Q-PEGL groups compared to the DN or PEGL group (P < 0.01). In addition, the FBG level in the Q-PEGL group was significantly lower than that in the Que group (P < 0.01). Compared with the Q-PEGL group, the increase of FBG induced by STZ in the PEGL, Que and DN groups was more pronounced, which peaked at week 4 and then gradually decreased. The peak of FBG in Q-PEGL group was at week 3. The FBG levels of all groups are shown in Fig. 2B.

At the end of the treatment period, the rHI and 24-h urinary protein were significantly lower in the NC group than those in the other groups (P < 0.01). In addition, the rHI and 24-h urinary protein were lower in the Q-PEGL and Que groups than those in the DN and PEGL groups (both *P* < 0.05), but there was no significant difference between the DN and PEGL groups (P > 0.05). The rHI and 24-h urinary protein were lower in the Q-PEGL than those in the Que group (*P* < 0.05). The rHI and 24-h urinary protein of all groups are shown in Fig. 2C,D, respectively.

### Changes in oxidative stress indicators in serum and kidney

**Figure 3:**
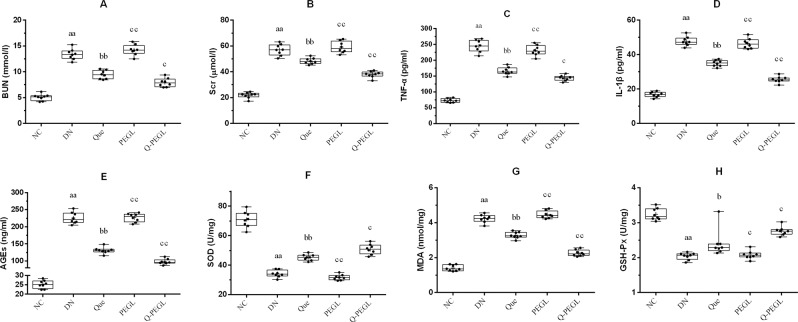
The mean serum and kidney chemistry of all groups (*n* = 8). (**A**) blood urea nitrogen. (**B**) serum creatinine. (C) tumor necrosis factor-α. (**D**) interleukin-1β. (**E**) advanced glycation end products. (**F**) superoxide dismutase. (**G**) malonaldehyde. (**H**) glutathione peroxidase. Data were presented as the mean ± S.D. ^a^*P* < 0.05, ^aa^*P* < 0.01 *versus* control group. ^b^*P* < 0.05, ^bb^*P* < 0.01*versus* DN group. ^c^*P* < 0.05, ^cc^*P* < 0.01 *versus* Que group. NC: normal control; DN: blank treatment; Que: free quercetin 50 mg/kg/day; PEGL: liposomes dissolved in 0.9% sodium chloride 150 mg/kg/day; Q-PEGL: Pegylated quercetin liposomes 200 mg/kg/day.

Figure 3 shows serum and kidney chemistry of the 5 groups of rats at the end of the treatment period. Levels of BUN, Scr, TNF-α, IL-1β, AGEs and MDA were lower in the NC group than those in the other groups (*P* < 0.01). The Que and Q-PEGL groups had lower levels of BUN, Scr, TNF-α, IL-1β, AGEs and MDA compared to the DN or PEGL groups (*P* < 0.05). There was no significant difference in these variables between the DN and PEGL groups (*P* > 0.05). Q-PEGL significantly decreased BUN, Scr, TNF-α, IL-1β, AGEs, and MDA (P < 0.05) compared to Que. NC group had higher levels of SOD and GSH-Px than the other groups (P < 0.01). Que and Q-PEGL groups had higher levels of SOD and GSH-Px (P < 0.05) compared to the DN and PEGL groups, and the effect of Q-PEGL was greater than Que (P < 0.05). There was no significant difference in these variables between the DN group and the PEGL group (P > 0.05).

### Histopathological of kidneys

**Figure 4:**
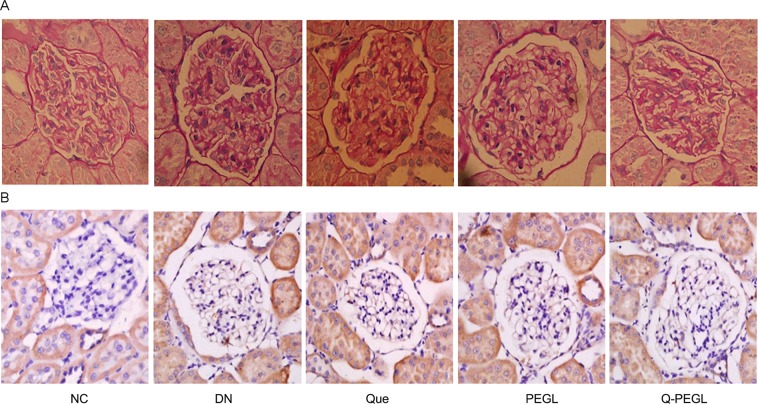
(**A**) Histopathological changes in PAS-stained sections were detected in all groups. The representative images are shown from different renal tissue sections from each group. Original magnification, 400×. (**B**) Representative photomicrographs of kidneys showing AGEs immunohistochemistry. The kidneys of DN rats showed highest intensity expression of AGEs. The kidneys of Q-PEGL rats showed lower expression of AGEs than other groups, except for comparison with NC group. Original magnification, 400×. NC: normal control; DN: blank treatment; Que: free quercetin 50 mg/kg/day; PEGL: liposomes dissolved in 0.9% sodium chloride 150 mg/kg/day; Q-PEGL: Pegylated quercetin liposomes 200 mg/kg/day.

Images of PAS-stained kidney tissue sections from selected experimental groups were obtained by using an optical microscope, as shown in Fig. 4A. The histological appearance of the glomerular and renal tubules were normal in the NC group. The DN and PEGL groups showed severe glomerular and tubular changes. These changes included glomerular volume atrophied, mesangial extracellular matrix increase, high deposition of glycogen, and basement membrane thickening. Q-PEGL and Que treatment alleviated the renal lesions, especially the Q-PEGL treatment. A significant increase in mesangial index was detected in the DN and PEGL groups compared to the NC group (*P* < 0.01 for both). The mesangial indices in the Que and Q-PEGL groups were significantly lower than that of the DN group (P < 0.05, P < 0.01).

### Renal immunohistochemistry

**Figure 5:**
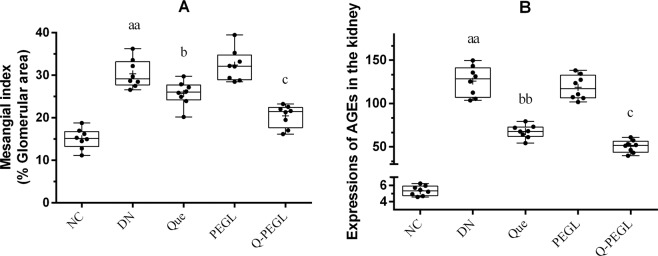
(**A**) Glomerular mesangial matrix expansion quantified from PAS staining. (**B**) Expression of AGEs in the tissues by immunohistochemistry at the end of the treatment period. Data were presented as the mean ± S.D. ^a^*P* < 0.05, ^aa^*P* < 0.01 *versus* control group. ^b^*P* < 0.05, ^bb^*P* < 0.01*versus* DN group. ^c^*P* < 0.05, ^cc^*P* < 0.01 *versus* Que group. NC: normal control; DN: blank treatment; Que: free quercetin 50 mg/kg/day; PEGL: liposomes dissolved in 0.9% sodium chloride 150 mg/kg/day; Q-PEGL: Pegylated quercetin liposomes 200 mg/kg/day.

The kidney AGE expression at week 8 by immunohistochemistry is shown in Figs. 4B and 5B. In the NC group, little immunofluorescence around the renal corpuscle wall and the tubular basement membrane was observed. Notably, AGEs expressions in the DN and PEGL groups were higher than those in the other groups (*P* < 0.01, for all), no significant difference was found between the DN and PEGL groups (*P* > 0.05). Interestingly, Que and Q-PEGL groups showed considerably decreased immunofluorescence labeling around the capsule and on the tubular basement membrane. The reduction of AGE expression in the Q-PEGL group was more pronounced than that in the Que group (P < 0.05).

## Discussion

Diabetic hypertension and unresolved inflammation in DN lead to renal fibrosis and kidney failure. Currently, the mainstays of DN treatment are glycemic control and blood pressure lowering using durgs to block the renin-angiotensin system. Despite emerging clinical candidates under investigation for the treatment of DN, there is a lack of successful new treatments for DN. Some innovative treatment make use of traditional anti-diabetic drugs (insulin and exenatide), cytokines, metabolic modulation, nucleotide and quercetin^16^. Quercetin is an active compound for anti-cancer, anti-bacterial, anti-allergic and analgesic, and improves DN through various signaling pathways, making it a promising compound for the management of these diseases^7,19^. The T2DM recovery from oxidative stress by quercetin was observed in single cell alkaline gel electrophoresis^5^. In this study, we showed quercetin carried by a synthesized PEGL was beneficial in reducing the deterioration of DN in rats.

Liposomes are non-toxic, biocompatible, biodegradable, and have the following advantages as carriers in drug delivery: first, liposomes can reduce toxicity; second, hydrophilic and lipophilic agents can be incorporated into liposomes; third, liposomes control the drug release; and fourth, the liposome delivery system increases bioavailability^12^. However, because quercetin has the disadvantage of low solubility *in vivo* and short cycle time, we synthesized a PEGL carrier to load quercetin to improve its biological activity *in vivo*^20,21^. The mass ratio of the composition of the Q-PEGL compound quercetin/lecithin/cholesterol/polyethylene glycol 4000 was 6:13:4:1 in weight ratio^9^, and the mass of the chloroform/methanol was very small. To ensure similar load of quercetin, Que (50 mg/kg) and Q-PEGL (200 mg/kg) were administered intragastrically in our study. In addition, PEGL (150 mg/kg) and Q-PEGL (200 mg/kg) were also administered intragastrically. Our results showed that Q-PEGL sustained a higher concentration of quercetin in plasma and kidney tissue compared to the free Que group after a given time point. The peak of quercetin appeared at 60 minutes, while the result of Yuan *et al*.^9^ was at 30 minutes, probably due to the Q-PEGL in our study was intragastrical administered instead of intravenous injection.

STZ is a natural compound that can induce DN through selectively destroying islet β cells of animals, because DN is usually associated with reduced viability and dysfunction of pancreatic β cells^23^. The rat DN model induced by STZ increased FBG and proteinuria in our study. Previous studies have shown that quercetin or quercetin liposomes were effective in controlling FBG, BWs, rHI, biochemistry and pathology in STZ induced diabetic animals^22^, which is consistent with our observation. Quercetin can reduce the plasma glucose level because it inhibits intestinal glucose absorption while enhances tissue and organ glucose levels;, quercetin increases insulin secretion by β-cells and protect DM against oxidative stress^7^. Administering quercetin to INS-1 pancreatic β-cells potentiated insulin secretion through enhanced glucose metabolism and protected β-cells against oxidative damages^24^. In STZ induced DN models of our study, quercetin liposomes or free quercetin were able to prevent body weight loss, decrease the renal hypertrophy index, lower blood glucose levels, and reduce the 24-h urinary protein. The effect of quercetin liposomes was stronger than quercetin alone. In addition, FBG peaked at 3 weeks and 4 weeks in the Q-PEGL and the Que group respectively, and in both groups, it decreased gradually with time. This difference in the peak time can be explained by the slow release of PEGL carrier. Increased kidney weight caused by diabetes indicates renal hypertrophy, a common finding in the early stages of diabetes^25,26^. In this study, interstitial fibrosis, mesangial contraction, and structural remodeling contributed to the decrease in renal hypertrophy, and improved renal function reduced the 24-h urinary protein.

The oxidative stress is considered to be important in the development of diabetic nephropathy^5,20,27^. Kuo *et al*.^28^ suggested that the interaction between ROS production and the anti-oxidant pathways can contribute to the progression of diabetic nephropathy. SOD, MDA, TNF-α and IL-1β are the most common indicators of oxidative stress. GSH-Px is one of the key enzymes that scavenge free radicals in the body. These factors can reflect the body’s antioxidant capacity. Literature shows that being an intracellular antioxidant, GSH-Px protects DNA, proteins and other biomolecules from ROS. AGEs are products of non-enzymatic glycosylation of proteins under chronic hyperglycemia, and levels of AGEs reflect the severity of renal injury. AGEs are thought to be a mechanism of action in the pathogenesis of clinical complications of diabetes, and accelerating synthesis and tissue deposition of AGEs leads to the microvascular alterations in DN^29^. Furthermore, in this study, DN model has shown that Que and Q-PEGL have the antioxidant capacity and scavenge free radicals, as evidenced by decreased levels of MDA, TNF-α, IL-1β, AGEs and increased activity of SOD, GSH-Px. Our results also revealed that Que and Q-PEGL significantly attenuated the increase in Scr, BUN levels, and reduced histopathological damage of kidneyin DN rats.

Long-term hyperglycemia increases secretion of inflammatory and fibrotic factors by renal cells, leading to cell hypertrophy and proliferation, and renal interstitial fibrosis^30^. In our study, pathological changes including glomerulosclerosis, interstitial fibrosis, and tubular atrophy due were observed, and these observation are consistent with previous studies in rats^30,31^. Xu *et al*.^32^ and Chen *et al*.^33^ have also reported that quercetin can improve the shape of the kidney, delay the progression of DN, and prevent diabetes-induced oxidative stress.

During hyperglycemia, the formation of hyperglycosylated AGEs in the glomerular basement membrane collagen is increased, which is extensively cross-linked with collagen, altering its spatial structure, increasing vascular permeability, mesangial proliferation and basement membrane thickening. The presence of AGEs in the kidneys of normal control rats indicates that AGEs occur even in healthy kidneys. Low levels of AGEs are probably resulted from enzyme-mediated detoxification^21^. Our results showed that quercetin liposomes and quercetin decreased the glomerular mesangial matrix expansion index and AGE levels, and the effect of quercetin liposomes was more significant. Gomes *et al*.^19^ showed that quercetin treatment can significantly improve renal structural changes, including glomerulosclerosis index and kidney/body weight normalization. The PAS and immunohistochemistry results showed that the pathological changes in the Q-PEGL group were better than those in the Que group. However, the relevant indicators for the PEGL group were similar to the DN group. This suggested that PEGL did not delay the progression of DN or prevent diabetes-induced oxidative stress. The study has limitations. We did not verify the solubility of Que and Q-PEGL, and the dose used in the study was based on previous study, which could introduce potential bias to the results.

In conclusion, the liposomes-loaded quercetin was successfully prepared using ratio of a mixture of quercetin/lecithin/cholesterol/polyethylene glycol 4000 and used for drug delivery by the intragastric administration route. *In vivo* study exhibited that the developed formulation had renal protective effects in STZ-induced DN rats by reducing oxidative stress, attenuating AGE expression, and delaying the progression of DN. In addition, the positive effect of quercetin liposomes on diabetic nephropathy is greater than that of quercetin alone. Therefore, quercetin liposomes can be used as a valuable therapeutic agent in DN.

## Materials and Methods

### Chemicals

High-purity lecithin of soybean, cholesterol, polyethylene glycol 4000, glucosidase, streptozotocin and quercetin were purchased from Sigma (MO, USA). Rat TNF-α and IL-1β ELISA kits were purchased from Elabscience Biotechnology Co. (Wuhan, China). The rat AGEs ELISA kit was purchased from Cusabio (Wuhan, China). All the other chemicals were purchased from Sigma (MO, USA). The SOD assay kit was purchased from Sigma-Aldrich (Shanghai, China).The MDA and GSH-PX assay kits were purchased from Elabscience Biotechnology Co. (Wuhan, China). All the other reagents used in the present study were of analytical grade and were purchared from Nanjing Jiancheng Bioengineering Institute (Nanjing, China).

### Animals

A total of 88 male Sprague-Dawley rats, weighing 190~220 g, were purchased from the Animal Laboratory of Guilin Medical University (Guangxi Province, China). The animals were maintained under standard laboratory conditions with a 12-h light:12-h dark cycle, at a temperature of 25 ± 2 °C, throughout the study. Animals were fed with standard rat chow and tap water *ad libitum* before and during the experiments. The animals were euthanized by injection of pentobarbital sodium (200 mg/kg) after the experiments. All study protocols including diabetes induction and sacrifice operation were approved by the ethics review board of the Institutional Animal Care and Use Committee at the Guilin Medical University. The animal experiment followed the guide for the care and use of laboratory animal published by the US National Institutes of Health (NI H Publication updated in 2011).

### Preparation of pegylated quercetin liposomes and empty pegylated liposomes

Pegylated quercetin liposomes (Q-PEGL) and empty pegylated liposomes (PEGL) were prepared at the pharmaceutical laboratory of Guilin Medical University (Guangxi Province, China). Briefly, a mixture of quercetin/lecithin/cholesterol/polyethylene glycol 4000 (6:13:4:1 by weight) was dissolved in a chloroform/methanol (3:1, v/v) solution, and then evaporated to dryness in a rotary evaporator^9^. The dried lipid films were sonicated in a 5% glucose solution in a constant container, then concentrated and lyophilized. The PEGL was prepared in the same manner as the Q-PEGL, which did not contain quercetin in the mixture. According to the calculation reported by Mukhopadhyay *et al*.^17^, the final Q-PEGL and PEGL are small unilamellar liposomes with a size range of 128.8 ± 18.05 nm. In addition, the Que encapsulation efficiency and load capacity were 87.1 ± 2.7% and 58 ± 7%, respectively. Q-PEGL and PEGL were concentrated, lyophilized under vacuum for 5 hours and stored at −20 °C. The final product has good solubility and can be used directly or intraperitoneally dissolved in saline^8^.

### Pharmacokinetic studies in free Que or Q-PEGL treated rats

The DM modle induction was as follows: forty-eight rats were fasted overnight and then given a single intraperitoneal injections of 60 mg/kg STZ dissolved in 10 mM citrate buffer (pH 4.5)^18^. Three days after STZ administration, the FBG was determined in a tail vein sample using blood glucose meter (LifeScan, CA, USA). Rats with FBG levels above 16.7 mM for 3 consecutive days were considered DM^13^. DM rats were fed with standard rat chow and tap water *ad libitum* for 4 weeks. All DM rats were transferred to metal metabolic cages and 24-hour urine samples were accurately collected.

A 24-hour urinary albumin excretion rate of >30 mg/24 h was observed at week 4, indicating the successful construction of DN^13^. Rats were treated intragastrically with free Que (50 mg/kg) or Q-PEGL (200 mg/kg) dissolved in 0.9% sodium chloride. Dose of 200 Q-PEGL and 50 Que mg/kg/day allows similar Que load between groups for the comparison according to our estimation. At each time point (5, 15, 30, 60, 120, 240, 720, and 1,440 mins), three rats were sacrificed and their blood was collected from the abdominal aorta. The plasma was obtained by centrifugation (12,000 × *g* for 20 min). The kidney tissue was excised, weighed and homogenized. Glucosidase was added into the plasma samples and incubated for 2 hours at 37 °C. Plasma and homogenized kidney tissue were extracted with ethyl acetate, and the supernatant was collected and evaporated to dryness. The dry residue was dissolved in methanol for high-performance liquid chromatography^9^. The level of quercetin in plasma or kidney is expressed as nmol/mL.

### Experimental design

There were 5 groups of rats. The DN model preparation was described above. DN rats were randomly divided into 4 groups (*n* = 8 per group), and treated intragastrically with quercetin liposomes (200 mg/kg/day, Q-PEGL group), free quercetin (50 mg/kg/day, Que group), liposomes dissolved in 0.9% sodium chloride (150 mg/kg/day, PEGL group), and blank treatment (DN group). Eight non-diabetic rats received sodium chloride and served as a normal control (NC group). Treatment of Q-PEGL, Que or PEGL was started since day 3 after STZ injection, at 9:00 a.m.–10:00 a.m. each day, and lasted for 8 weeks.

Rat urine samples were collected weekly with a metabolic cage during drug treatment and were purified by centrifugation at 3000 *g* for 5 min to remove the impurities. At the end of the experiment, a 24-h urine sample was collected, and then a further 12 h fast, blood sample was taken from the abdominal aorta under an anesthesia of intraperitoneal administration of 50 mg/kg 1% chloral hydrate. The serum sample was centrifugation at 3 000 g for 5 minutes at 4 °C. The 24-h urine samples and serum were stored at −80 °C until analysis. Rats were sacrificed following the above steps. Both kidneys from each rat were removed and weighed. The right kidney was divided into two parts and stored in 10% formaldehyde solution for periodic acid-Schif (PAS) staining and immunohistochemical assays. The left kidneys were stored at −80 °C for biochemical evaluation.

### Evaluation of BWs, FBG, rHI, and 24-h urinary protein

Body weights and fasting blood glucose levels were recorded each week before and after STZ induction, including drug intervention period. FBG was measured by a blood glucose meter (LifeScan, CA, USA) in samples obtained from the tail vein. It was about 2 mins from blood collection to obtaining results. The renal hypertrophy index (mg/g) was calculated as follows: renal index = kidney weight (mg)/body weight (g). 24-h urine protein was determined on an AU2700 automatic biochemical analyzer (Olympus, Japan).

### Evaluation of serum BUN, Scr, TNF-α, IL-1β and AGEs levels

Serum was centrifuged at 3600 *g* for 10 minutes and the supernatant was then used to assess the renal function. The levels of BUN and Scr were determined on an AU2700 automatic biochemical analyzer (Olympus, Japan). The levels of TNF-α, IL-1β and AGEs were measured according to the manufacturer’s instructions of the immunoassay kit. The assay employs the ELISA techniques.

### Evaluation of SOD, MDA and GSH-Px in kidney

Approximately 100 mg of kidney tissue was homogenized in 5% phosphate-buffered saline using a homogenizer. The kidney homogenate was then centrifuged at 15000 *g* for 10 minutes at 4 °C and the clear supernatant was collected. The SOD value (U/mg) was measured by the xanthine oxidase activity assay kit; the MDA level (nmol/mg) was measured by the thiobarbituric acid method (MDA colorimetric assay kit); and the GSH-Px value (U/mg) was measured using a colorimetric assay kit according to the manufacturer’s instructions (Elabscience Biotechnology Co., China).

### Histopathological staining

A portion of the right kidney was trimmed into small pieces and stored in phosphate buffered 10% formaldehyde solution for 24 hours. After embedding these pieces in paraffin, a thin slice of 5 mm thickness was cut. Glomerular damage was assessed in PAS stained sections. Ten glomeruli randomly selected from each group were quantified in PAS stained sections using image analysis software (Image-Pro plus 6.0) to assess the percentage of PAS-positive areas in the glomerulus, which was expressed as mesangial index.

### Immunohistochemical measurement of AGEs

The other part of right kidney tissues was fixed, dehydrated, paraffin embedded, and cut into paraffin slices (4 mm). Tissue sections were deparaffinized, rehydrated, washed in water and subjected to antigen retrieval (60 °C, 2 hours) in 0.1 M citrate buffer (pH 6.0). After blocking, the selected sections were incubated with AGEs (1:100) for 1 hour. After washing with PBS, the sections were incubated with goat anti-rabbit IgG (1:100) for 20 minutes. Finally, the nuclei were stained with DAB, washed with PBS and mounted in a fluoromount solution. Incubation with immunoglobulin of the same species at the same concentration with no primary antibody was used as a negative control. Five fields of view were digitized using a 400× magnifying objective. The sum of IOD of all fields was collected and analyzed by Image-Pro Plus 6.0.

### Statistical analysis

Data were analyzed by the statistical software IBM SPSS version 23 (Chicago, IL, US). Values are presented as the means ± standard deviation. For comparisons in multiple groups, one-way analysis of variance (ANOVA) was used and post hoc analysis was done using Bonferroni test. *P* < 0.05 was considered as statistically significant for a two-tailed test.
